# Harnessing beta-cell replication: advancing molecular insights to regenerative therapies in diabetes

**DOI:** 10.3389/fendo.2025.1612576

**Published:** 2025-06-19

**Authors:** Rupangi C. Vasavada, Sangeeta Dhawan

**Affiliations:** Department of Translational Research and Cellular Therapeutics, Arthur Riggs Diabetes and Metabolism Research Institute, City of Hope, Duarte, CA, United States

**Keywords:** beta cells, replication, proliferation, regeneration, therapeutics, diabetes

## Abstract

Diminished functional beta-cell mass is a key pathogenic mechanism underlying both type 1 and type 2 diabetes (T1D and T2D), precipitated by the progressive impairment of insulin secretion, loss of cellular identity, and ultimately, beta-cell death. The replenishment of beta-cell deficit through the transplantation of pancreatic islets from cadaveric donors or beta-cells derived from human embryonic stem cells has shown transformative therapeutic potential. However, the regeneration of functional beta-cell mass *in vivo* remains an important therapeutic goal, as a more physiological and scalable approach. Effective beta-cell replenishment must address the underlying causes of beta-cell loss, such as cellular stress and autoimmunity, while simultaneously promoting beta-cell regeneration, function, and survival. Advances in the mechanistic underpinnings of beta-cell differentiation, growth, and survival, coupled with cutting-edge high-throughput screening methods have accelerated the discovery of novel therapeutic targets and small-molecule interventions. Current strategies for *in vivo* beta-cell expansion include modulating the cell-cycle to promote replication, reprogramming non-beta-cell lineages into beta-cells, and enhancing beta-cell survival. However, the limited regenerative capacity and inherently high stress sensitivity of beta-cells pose significant barriers to their *in vivo* expansion, further complicated by the fundamental conflict between replication and functional maintenance, and the high vulnerability of replicating cells in a metabolically stressed environment. There has been tremendous progress in developing approaches that simultaneously promote beta-cell expansion and function. In this review, we discuss the recent advances in beta-cell expansion, along with remaining challenges and emerging opportunities to address them.

## 1 Introduction

Strategies that promote the regeneration of functional beta-cells *in vivo* by stimulating their proliferation represent an attractive and physiological therapeutic approach that can benefit patients with T2D as well as T1D, given that residual beta-cells persist in established T1D. Human beta-cell expansion poses a unique therapeutic challenge due to their limited replicative capacity, heightened vulnerability to stress during proliferation, and remodeling of beta-cell heterogeneity throughout diabetes progression. Here, we examine advances in stimulating beta-cell expansion, focusing on how recent mechanistic insights into beta-cell biology are informing the development of therapies that can simultaneously enhance proliferation, function, and survival in diabetes.

## 2 Targeting beta-cell proliferation for regeneration of beta-cell mass

Targeting the pathways involved in beta-cell proliferation has emerged as a therapeutically promising approach for beta-cell expansion *in vivo*, leveraging one of the body’s natural mechanisms for growth in early life as well as subsequent adaptive beta-cell expansion in response to increased insulin demand. Many of the pathways regulating beta-cell proliferation are amenable to small molecule therapeutics, making this approach particularly attractive for clinical translation. Consequently, significant efforts have focused on identifying the molecular control of beta-cell replication.

### 2.1 Harnessing physiological control of beta-cell replication for regeneration

**Figure 1:**
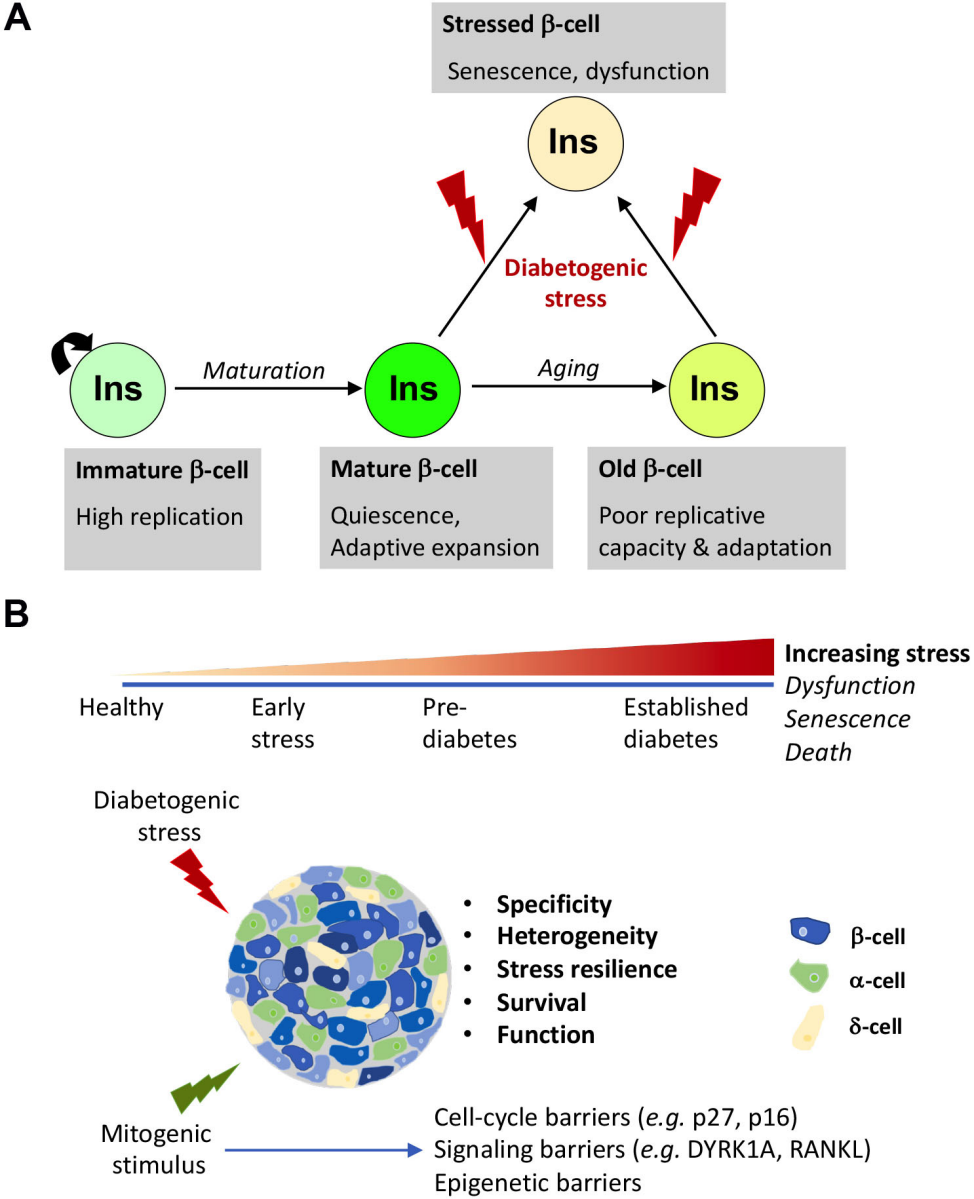
Beta-cell proliferation: natural history and therapeutic considerations. **(A)** Physiological control of beta-cell expansion in health and disease. The beta-cell proliferation landscape changes substantially throughout life. Early in postnatal life, beta-cells rapidly expand to accommodate growth and establish beta-cell mass. As growth gradually tapers out, beta-cells exit cell-cycle and assume a functionally mature and quiescent state, which retains the capacity to expand on demand, in response to physiological challenges for increased insulin needs, such as pregnancy, obesity, islet injury etc. This capacity of adaptive replication, however, declines with aging due to the onset of p16 accumulation dependent replicative senescence. The replicative response of beta-cells not only depends on age but also varies depending on the nature of the metabolic demand. Continuously high metabolic demand and/or exposure to inflammation can trigger maladaptation and result in dysfunction and a state of permanent cell-cycle exit marked by a pro-inflammatory phenotype: stress-induced senescence. If the stress persists, beta-cells can eventually succumb and undergo cell-death. Ultimately, this can result in diabetes. **(B)** Factors influencing the efficacy of mitogenic agents on beta-cell expansion in diabetes. The beta-cell phenotype evolves through the course of the initiation and progression of diabetes, amounting to significant heterogeneity of disease. To promote beta-cell expansion in these conditions, the therapeutic agents must not only overcome barriers to beta-cell replication that are especially stringent in human beta-cells, but must also promote resilience to stress and survival, support optimal insulin secretion post-replication, and ensure a healthy milieu by combating inflammation and/or promoting optimal islet niche. This can be achieved either by using mitogenic agents that inherently support beta-cell health or by combining them with therapeutic agents that resolve stress and boost function. Ideally, an optimal therapeutic agent must also selectively target beta-cells to avoid off-target effect. Finally, there is considerable heterogeneity in beta-cell phenotypes, which is further remodeled in disease. Identifying which beta-cell subset maybe most responsive for expansion remains an ongoing line of enquiry that will benefit approaches that involve replication to boost beta-cell mass in diabetes.

Replication is the primary mechanism of postnatal beta-cell growth and crucial for adaptation to metabolic challenges (1–3) (**Figure 1A**). Understanding the physiological regulators of replication can reveal potential targets for therapeutic beta-cell expansion in diabetes. Beta-cells undergo substantial replication during early postnatal stages to establish beta-cell mass (4–6). Proliferation gradually declines as beta-cells mature functionally, revealing an inverse relationship between function and replication (7–9). The mTOR/PI3K/Akt pathway integrates nutrient cues and growth signals to promote early postnatal beta-cell expansion, while increased AMPK (AMP-activated protein kinase) activity subsequently enhances functional maturation while restraining proliferation through cell-cycle regulation (10–12). The calcineurin/NFAT pathway is another critical regulator of beta-cell growth and functional maturation, with DYRK1A kinase inhibiting the pro-proliferative activity of NFATs and serving as a cell-cycle brake (13–15).

The regulation of postnatal beta-cell replication depends on the balance between mitogen-induced cyclin D2-Cdk4 complex formation and inhibitory actions of cyclin kinase inhibitors (CKIs) on the cyclinE-Cdk2 complexes (1, 16–18). As growth progresses, accumulation of the CKI p27 drives cell-cycle exit and a quiescent beta-cell state (19). Beta-cell mass is largely established following the postnatal proliferative phase, with subsequent maintenance occurring primarily through limited replication and survival (6, 20, 21). Multiple mechanisms reinforce beta-cell quiescence: p27 degradation control via SCF ubiquitin ligase and Menin-mediated epigenetic regulation, which maintains CKI expression and co-represses transcription of cell-cycle genes driven by TEAD1, an effector of the Hippo pathway which controls tissue growth (22–25). p27 degradation is essential for proliferation of mature beta-cells in response to increased insulin demand, such as in obesity and pregnancy. The metabolic state of beta-cells is a key determinant of proliferative capacity (26). The postnatal maturation transforms beta-cell proliferative response from growth-driven mass expansion to selective compensatory proliferation upon increased insulin demand (27). However, this capacity diminishes with age due to replicative senescence marked by accumulation of CKI p16 (28–32). This age-related decline involves altered epigenetic control, with young beta-cells repressing p16 through Polycomb proteins (Ezh2, Bmi1) (33, 34). Aging reduces Ezh2 levels and increases Trithorax complex (containing Mll1 and JmjD3) activity to induce p16 accumulation (35). DNA methylation is another epigenetic mechanism that modulates the transcriptional programs involved in beta-cell maturation and proliferation (36). Age-dependent shifts in growth factor signaling drive beta-cell senescence: declining PDGF signaling reduces Ezh2 expression, while increased TGFβ signaling activates Trithorax-dependent p16 accumulation, promoting replicative senescence (37, 38).

Our understanding of pathways that modulate physiological beta-cell expansion has revealed species-, age-, and physiological state-specific differences in replicative potential and identified several therapeutic avenues. For instance, PDGF can stimulate β-cell proliferation only in juvenile human islets, while more downstream manipulation of the PDGF/Ezh2 pathway using beta-cell specific overexpression of Ezh2 can overcome the age-related replicative barrier in mice (35, 37). Similarly, TGFβ inhibitors can successfully drive the expansion of both adult murine and human beta-cells (38). Along these lines, antibody arrays on serum of young mice identified Wnt-1 inducible signaling protein 1 (Wisp1) as a circulating factor that can induce rodent and human beta-cell replication (39). Compensatory beta-cell expansion during insulin resistance (IR) involves critical growth factors produced by liver (40). Proteomic analysis of liver secretome under conditions of IR has identified the hepatocyte-secreted protease inhibitor SerpinB1 as a key factor promoting beta-cell replication through elastase inhibition—an effect that can be mimicked by small molecule elastase inhibitors. In agreement with this, SerpinB1 deficient mice display poor beta-cell compensation in response to IR (41). Pregnancy hormones have also been explored as therapeutic agents for beta-cell expansion. Prolactin (PRL), placental lactogen (PL), estrogen, and other factors such as serotonin—naturally upregulated during pregnancy—induce beta-cell replication, providing another physiology-based approach to beta-cell regeneration (42–45). PRL-dependent beta-cell replication *in vivo* requires the protein osteoprotegerin (OPG), as PRL fails to induce beta-cell expansion in whole-body OPG knockout mice (46). Notably, while PRL can only enhance beta-cell replication in rodents but not in human islets due to the lack of PRL-receptors (47), OPG can independently induce both rodent and human beta-cell replication *in vitro* and *in vivo* (46). Glucagon-like peptide 1 (GLP1) is another circulating factor that promotes beta-cell replication in mice, with GLP1 receptor agonists (GLP1RAs) emerging as potential therapeutic agents when used in combination with other proliferative agents capable of enhancing human beta-cell replication (48–50). Extracellular-vesicles (EVs) have emerged as critical modulators of cellular function in both health and disease, including diabetes (51–53). Recent work suggests that EVs derived from sources that naturally stimulate beta-cell replication and regeneration—such as stem and progenitor cells, and serum from young or pregnant individuals— may hold promise for beta-cell expansion (54).

### 2.2 Barriers to human beta-cell proliferation: lessons from islet pathologies

Much of our mechanistic understanding of beta-cell replication comes from rodent models, which exhibit higher baseline proliferation and mitogen responsiveness, limiting translational relevance. Mature human beta-cells display remarkable resistance to proliferation, presenting a significant challenge for regenerative approaches (55–57). The cell-cycle machinery differs substantially between species—humans have abundant CDK6 but minimal Cyclin D2, while mice show the reverse pattern (58). While replication is the primary mechanism of postnatal beta-cell growth in both mice and humans, its role in human beta-cell adaptive expansion has been debated (1–3, 59). Pathological conditions exhibiting abnormal beta-cell expansion have provided valuable insights into the molecular barriers that normally restrict human beta-cell replication. Comprehensive genomic, epigenomic, and transcriptional profiling of human insulinomas—rare beta-cell tumors characterized by insulin overproduction—has provided crucial insight into human beta-cell proliferation. Most insulinomas exhibit concurrent mutations in multiple chromatin regulators, particularly in the Polycomb and Trithorax Group genes (such as *EZH2*, *YY1*, *RING1*, *BMI1*, *MEN1*, *KMT2C* and *KDM6A*). EZH2 overexpression appears in most insulinomas, likely driving hyperproliferation and altered gene expression (60). All insulinomas share an aberrant DNA methylation signature within the 11p15.5-p15.4 sub-region containing critical imprinting control regions for the *IGF2/H19* and *KCNQ1/CDKN1C* loci that regulate body growth (61). Mutations in this region cause Beckwith-Wiedemann syndrome, another condition with beta-cell hyperproliferation (62). Comparison of normal beta-cell and insulinoma transcriptomic profiles has revealed the DREAM (dimerization partner, retinoblastoma-like, E2F and MuvB) repressor complex as a key transcriptional modulator of human beta-cell proliferation. DREAM complex assembly occurs in response to DYRK1A activation to establish and maintain quiescence. Loss of DYRK1A results in the reorganization of DREAM components into a pro-proliferative complex called MMB (MuvB complex with MYBL2) which promotes entry to the S phase (63). Indeed, DYRK1A inhibition has emerged as a major target for inducing human beta-cell proliferation.

### 2.3 Unbiased approaches for beta-cell expansion

Increasing efforts are focused on unbiased identification of molecules that promote beta-cell expansion with higher efficacy and cell specificity, mitigating off-target effects. These efforts include high-throughput screens (HTS) utilizing human islet cells to screen chemical and RNAi libraries (64–68). Among the most notable advances from these efforts is the identification of the dual-specificity tyrosine-regulated kinase-1a (DYRK1A) as a central negative regulator of beta-cell proliferation. DYRK1A intersects with multiple arms of beta-cell replication machinery, including NFATs and DREAM complex (13, 63). Multiple independent screens converged on DYRK1A, leading to the discovery of Harmine and INDY, small molecules that induce beta-cell replication through DYRK1A inhibition (69). Parallel screens identified aminopyrazine analogs and the adenosine kinase inhibitor 5-iodotubercidin (5-IT) as additional compounds that enhance human beta-cell replication through DYRK1A/NFAT-dependent and -independent mechanisms (70, 71). Another screen leveraged a VGF promoter-reporter to screen for NKX6.1 pathway activators, based on findings that NKX6.1 enhances beta- but not alpha-cell replication and induces VGF expression, discovering a compound that selectively promotes human beta-cell proliferation via a mechanism distinct from DYRK1A inhibition (72).

Complementing these *in vitro* approaches, zebrafish has emerged as a scalable *in vivo* screening model system, offering real-time assessment of islet expansion and function. A luminescence-based ubiquitination reporter screen in zebrafish identified an inhibitor of salt-inducible kinases (SIKs) as a potent cross-species stimulator of beta-cell proliferation via unfolded protein response (UPR) activation (73). The relevance of SIKs was independently validated in an RNAi screen of the human G protein-coupled receptor (GPCR) family on human islets, which uncovered GPR3 as a negative regulator of human beta-cell proliferation via modulation of SIK2 activity (74). Similarly, a small molecule screening in zebrafish identified the non-canonical IκB kinase TANK-binding kinase 1 (TBK1) as a negative regulator of beta-cell replication and shown to directly modulate rodent and human beta-cell regeneration (75).

An unbiased search using microarrays identified OPG as a downstream effector of lactogens in the beta-cell. OPG induces both rodent and human beta-cell replication by inhibiting the Receptor Activator of NF-κB (RANK)/RANK ligand (RANKL) pathway (46, 76). This discovery led us to investigate Denosumab, a monoclonal antibody against human RANKL and an FDA-approved osteoporosis drug. Like OPG, Denosumab enhances human beta-cell replication *in vitro* and *in vivo* in human islets transplanted into immunodeficient mice (46, 76). Notably, Denosumab is currently in a phase1/2 multi-center clinical trial (NCT06524960) to assess safety and efficacy in improving beta-cell function in early T1D. More recently, we identified Leucine-rich repeat-containing G-protein coupled receptor 4 (LGR4) as a novel physiological inhibitor of the RANK pathway (77). The soluble extracellular domain of LGR4 (LGR4-ECD) has emerged as a potential new therapeutic for osteoporosis, revealing another promising target for enhancing beta-cell mass in diabetes (78).

## 3 Considerations for therapeutic expansion of beta-cell mass in diabetes

Beta-cell dysfunction and loss characterize both T1D and T2D, making beta-cell expansion an attractive therapeutic goal. However, effective translation of such candidates to clinical application requires careful consideration of other disease relevant aspects. First, the ideal therapeutic must not only enhance beta-cell replication but also improve beta-cell function and resilience. The heterogeneous nature of diabetes pathogenesis coupled with intrinsic beta-cell heterogeneity poses additional challenges, as therapeutic response could vary based on disease stage and corresponding changes in beta-cell phenotype. Additionally, specific targeting to beta-cells is essential to prevent off-target effects and minimize risks of cellular transformation. Successful beta-cell regenerative therapies must overcome these gaps.

### 3.1 Non-replicative mechanisms in beta-cell expansion: clues for combination therapy

Replication inherently creates cellular vulnerability, especially under diabetic stress conditions (79–81). Replication is also mutually incompatible with glucose-stimulated insulin secretion, as these processes compete for cellular resources and involve different metabolic states (7). Effective beta-cell expansion strategies in diabetes must therefore concurrently promote stress resilience and survival, while ensuring that cells can regain their mature phenotype and functional capacity following replication. Emerging evidence from pre-clinical studies shows that many therapeutic agents can simultaneously enhance human beta-cell replication while improving survival, function and/or maturity. Examples include inhibitors of DYRK1A and the RANK pathway that simultaneously promote beta-cell survival alongside expansion (49, 76, 82). These agents offer a distinct advantage for clinical applications, as they confer multiple beneficial effects on beta-cell health under disease-relevant stressors. Modulation of these pathways likely exerts temporally distinct effects on beta-cells, initially boosting survival and resilience, followed by successful expansion and restoration of function. Recent work on the TMEM219-IGFBP3 axis illustrates this perfectly; the ligand IGFBP3, markedly elevated in sera from both T1D and T2D patients, triggers beta-cell apoptosis by activating its cognate death receptor TMEM219. Pharmacological disruption of this axis using the extracellular domain of TMEM219 (ecto-TMEM219) provides temporally distinct benefits in preclinical diabetes models by initially enhancing beta-cell survival and preventing diabetes onset and promoting beta-cell expansion in the long-term (83).

Recent studies point to an even more complex mechanistic landscape, suggesting that some beta-cell proliferative agents may also induce trans-differentiation of non-beta-cells to beta-cells alongside replication. Single-cell transcriptomic analysis of human islets reveals that treatment with DYRK1A inhibitors targets cycling alpha cells which may transdifferentiate into beta-cells (84). This process could be particularly relevant in T1D, which is characterized by severe beta-cell depletion and widespread senescence among residual beta-cells, thus significantly limiting the pool of replication-competent beta-cells. The islet microenvironment also impacts beta-cell response to proliferative agents, with some molecules showing anti-inflammatory and immunomodulatory effects that create a more permissive niche for regeneration (76, 82).

Given this complexity, approaches that strategically combine agents that target complementary pathways could offer superior outcomes. Combining agents that enhance survival, mitigate ER-stress, or reduce inflammation with mitogenic stimuli can create synergistic effects for sustainable beta-cell expansion. This approach has been demonstrated using DYRK1A inhibitors with GLP-1RAs and TGF-β inhibitors, and combinations of sitagliptin with melatonin (49, 82, 85, 86). GLP-1RAs, widely used in T2D, offer multiple benefits including beta-cell regeneration (87, 88). Selective elimination of senescent beta-cells prevents immune-mediated destruction in T1D models and protects against T2D, with potential to transform a stress-prone environment into one conducive to both replication and trans-differentiation (89, 90). Similarly, pharmaceutical agents targeting the integrated stress response (ISR) have shown promise for enhancing beta-cell survival and function (91–94). Combining agents that target senescence or ISR with those directed at beta-cell proliferation may therefore be an effective way to removing barriers to regeneration in the setting of diabetes while preserving the regenerative potential of remaining healthy cells. Notably, IGFBP3 is a component of beta-cell senescence associated secretory phenotype (SASP) in T1D (89). Thus, targeting of IGFBP3/TMEM219 axis might offer additional protective effects against senescence (83).

Beyond these considerations, achieving cellular specificity remains a critical hurdle for clinical translation. Several innovative approaches are being developed to address this challenge, including leveraging beta-cell-enriched receptors such as GLP1R for drug targeting, engineering sophisticated nanoparticle and recombinant adeno-associated virus (rAAV) delivery systems, and repurposing existing FDA-approved medications with established safety profiles (46, 76, 82, 95–97). For instance, Denosumab has now advanced to early clinical trials (NCT06524960) following promising pre-clinical evidence of beta-cell regeneration. Similarly, a recent phase-1 safety study demonstrated that orally administered Harmine at doses below 2.7 mg/kg produced minimal to no adverse events in healthy volunteers, representing an encouraging step toward clinical application (98). Therapeutic targeting of pathways that are largely beta-cell specific, *e.g.* the IGFBP3/TMEM219 axis (83), would be crucial to improve specificity. These advances in targeting specificity, combined with approaches addressing overall beta-cell health, provide a comprehensive framework for developing therapeutically viable beta-cell regeneration strategies with acceptable safety profiles.

### 3.2 The proliferative beta-cell: what, when, where?

A fundamental question in developing therapeutic beta-cell expansion strategies concerns how regeneration may proceed across the heterogeneous pancreatic landscape in diabetes (99–104). For instance, would regeneration in T1D primarily occur in regions with residual beta-cells, in areas completely devoid of beta-cells, or both? Immune infiltration adds additional complexity—regions with active autoimmunity may not be permissive for regeneration despite having residual beta cells, while areas where inflammation has resolved might permit replication even with fewer remaining cells. There is growing recognition of beta-cell heterogeneity, with distinct subpopulations emerging throughout development, aging, and across islet regions, exhibiting variations in molecular characteristics, functional properties, and replicative potential (105–116). For instance, in mice, cells expressing Flattop (Fltp) display a functionally mature, post-mitotic phenotype, while Fltp- cells represent the proliferative beta-cell subset (117). Similarly, CyTOF mass cytometry revealed a small subpopulation of Ki67+ beta-cells in human pancreas (118), suggesting that replicative heterogeneity marks both rodent and human beta-cells. This is particularly relevant for diabetes therapies, where identifying target beta-cell subpopulations with high functionality and replicative potential is crucial.

Beta-cell heterogeneity undergoes significant temporal remodeling in response to metabolic stress during diabetes progression. Transition to a replication-permissive state represents one of the earliest responses to acute beta-cell stress, along with functional compensation and activation of stress-response and pro-survival pathways (119–122). Select beta-cell subpopulations in diabetes recapitulate molecular features of the more proliferative and immature neonatal beta-cells (116, 123–126), suggesting these subsets could be optimal targets for expansion. Emerging evidence shows that modest endoplasmic reticulum stress (ER stress) drives beta-cell replication in response to increased insulin demand, with beta-cells exhibiting an active unfolded protein response (UPR) showing more proliferative capacity (127). However, unresolved stress can eventually prevent cell-cycle progression, instead inducing a pro-inflammatory senescent state (99, 128).

Multiple studies have demonstrated the accumulation of senescent beta-cell subpopulations in both T1D and T2D, highlighting heterogeneous beta-cell proliferative capacity in diabetes (89, 90, 99, 105, 129, 130). Unrelenting stress in diabetes can cause irreversible cellular damage and cause cell-death. Agents that successfully promote beta-cell replication in diabetes must therefore simultaneously be able to mitigate stress, creating optimal milieu for expansion. Emerging data suggests that T2D islets exhibit enhanced responsiveness to proliferative agents. Interestingly, Wang et al. demonstrated that the rare Ki67+ beta-cells detectable in non-diabetic human pancreas were absent in T2D (118), supporting the hypothesis that diabetic conditions impair the ability of beta-cells to progress through cell-cycle. This apparent contradiction may reflect different phases of the disease, with early stress responses enhancing proliferative potential while chronic stress ultimately blocks cell-cycle progression.

Heterogeneity of beta-cell replicative potential and the subtype shifts observed in diabetes raise several questions (**Figure 1B**). First, which beta-cell subpopulations represent optimal targets for therapeutic expansion? Second, as most therapeutic candidates are tested in human islets from non-diabetic donors, question remains whether these interventions would be effective in diabetic patients. Finally, what impact does ongoing beta-cell expansion have on islet function? Addressing these questions requires understanding how beta-cell states and their replicative potential are dynamically modulated in response to stress. Defining temporal evolution of these populations can determine the optimal disease stage for therapeutic intervention. Advances in whole-pancreas visualization permit comprehensive temporal assessment of beta-cell expansion following drug treatment in pre-clinical models (82, 131). Using single-cell and spatial transcriptomic and proteomic approaches to map molecular trajectories of beta-cell functional and proliferative states at different disease stages will help decipher the optimal therapeutic window and target beta-cell population(s) to boost beta-cell expansion (106, 118, 132–136).

## 4 Next-Generation approaches for beta-cell regeneration

The future of beta-cell expansion approaches is rapidly evolving toward combinatorial strategies and precision therapies, warranting platforms that enhance target discovery and therapeutic specificity. Gene-editing platforms such as CRISPR have emerged as transformative tools enabling targeted modulation of gene expression, high-throughput screens, and disease modeling (137, 138). Recent proof-of-principle work has leveraged gene-editing tools to induce human beta-cell expansion by altering the epigenetic state and expression of specific cell-cycle modulators (139, 140). Recent work has also demonstrated the feasibility of using CRISPR to target regulatory genomic regions in primary human islets to modulate gene expression and islet function, an important advancement considering the challenges of delivering CRISPR components into quiescent, post-mitotic cells like human beta-cells (141, 142). Genome-wide CRISPR screens have also been used to identify critical regulators of beta-cell function and immune vulnerability (143, 144). Translating these findings to clinical applications requires careful validation in human beta-cells. Advances in long-term culturing of live pancreatic tissue slices now allow study of human beta-cells within their native microenvironment (145). This platform enables *in situ* lineage tracing to monitor beta-cell formation in real-time, assess therapeutic candidates in disease context, and evaluate off-target effects alongside islet function (132, 146). These technologies offer unprecedented opportunities to develop personalized regenerative approaches for diabetes, potentially transforming treatment paradigms.
